# A comparison of multiple imputation methods for missing data in longitudinal studies

**DOI:** 10.1186/s12874-018-0615-6

**Published:** 2018-12-12

**Authors:** Md Hamidul Huque, John B. Carlin, Julie A. Simpson, Katherine J. Lee

**Affiliations:** 1 Clinical Epidemiology and Biostatistics Unit, Murdoch Children’s Research Institute, Parkville, VIC 3052 Australia; 2 Department of Paediatrics, The University of Melbourne, Parkville, VIC 3052 Australia; 3 Centre for Epidemiology and Biostatistics, Melbourne School of Population and Global Health, The University of Melbourne, Parkville, VIC 3052 Australia

**Keywords:** FCS, Joint modelling, MICE, Multiple imputation, Multilevel multiple imputation, Longitudinal missing data, Linear mixed model

## Abstract

**Background:**

Multiple imputation (MI) is now widely used to handle missing data in longitudinal studies. Several MI techniques have been proposed to impute incomplete longitudinal covariates, including standard fully conditional specification (FCS-Standard) and joint multivariate normal imputation (JM-MVN), which treat repeated measurements as distinct variables, and various extensions based on generalized linear mixed models. Although these MI approaches have been implemented in various software packages, there has not been a comprehensive evaluation of the relative performance of these methods in the context of longitudinal data.

**Method:**

Using both empirical data and a simulation study based on data from the six waves of the Longitudinal Study of Australian Children (*N* = 4661), we investigated the performance of a wide range of MI methods available in standard software packages for investigating the association between child body mass index (BMI) and quality of life using both a linear regression and a linear mixed-effects model.

**Results:**

In this paper, we have identified and compared 12 different MI methods for imputing missing data in longitudinal studies. Analysis of simulated data under missing at random (MAR) mechanisms showed that the generally available MI methods provided less biased estimates with better coverage for the linear regression model and around half of these methods performed well for the estimation of regression parameters for a linear mixed model with random intercept. With the observed data, we observed an inverse association between child BMI and quality of life, with available data as well as multiple imputation.

**Conclusion:**

Both FCS-Standard and JM-MVN performed well for the estimation of regression parameters in both analysis models. More complex methods that explicitly reflect the longitudinal structure for these analysis models may only be needed in specific circumstances such as irregularly spaced data.

**Electronic supplementary material:**

The online version of this article (10.1186/s12874-018-0615-6) contains supplementary material, which is available to authorized users.

## Background

Longitudinal studies, where information on the same participants is obtained repeatedly over time, are frequently used in clinical and population health research. Analysis of data obtained from such studies is often impeded by the presence of missing data due to item or visit non-response and loss to follow-up [1, 2]. Commonly used analytic approaches exclude patients or records with missing data, which may lead to biased estimates and considerable loss of precision [3, 4].

Multiple imputation (MI) has become a very popular tool for dealing with missing data in recent years [5, 6]. MI involves the generation of multiple copies of the dataset in each of which missing values are replaced by imputed values sampled from their posterior predictive distribution given the observed data. Each completed dataset is analysed using the statistical model appropriate to the epidemiological question of interest, and the resulting estimates and standard errors are combined using Rubin’s rules [4]. MI methods generally assume data are missing at random (MAR), which requires that the probability of data being missing, conditional on observed data, is independent of the missing data.

Two general approaches for imputing missing data in the presence of multiple incomplete variables are available in standard computer packages [7–9]: MI based on the joint posterior distribution of incomplete variables, often referred to as joint modelling (JM), and fully conditional specification (FCS; also known as sequential regression and MI using chained equations (MICE)) [10–13]. The JM approach commonly assumes that the incomplete variables follow a multivariate normal distribution, often referred to as multivariate normal imputation [12]. FCS, on the other hand, imputes missing values using univariate conditional distributions for each incomplete variable given all the others, cycling iteratively through the univariate imputation models [13].

Both of these MI approaches were originally proposed for cross-sectional data but can be used to impute longitudinal data collected at equal intervals by considering repeated measurements of time-dependent variables as distinct variables [14], denoted as JM-MVN and FCS-Standard, respectively. Although relatively straightforward to implement using existing software, these methods (i) cannot accommodate longitudinal time-dependent covariates that are measured at irregular time intervals and (ii) may experience non-convergence due to model over-fitting and/or collinearity with large numbers of repeated measurements. Several extensions of the standard JM and FCS approaches for imputing cluster/longitudinal data have been proposed in the literature over recent years [14–20]. The extensions include limiting the number of time-dependent variables in the univariate imputation models within FCS [20]; and specifying imputation models based on the generalized linear mixed-effects model (GLMM) [14–19]. The GLMM-based approaches are generally based on more restrictive assumptions about modelling the correlation structure than the JM-MVN and FCS-Standard as these approaches allow for arbitrary dependence of each variable on other variables. The GLMM-based approaches also use the observation at a given time-point as the unit of analysis rather than the individual. It is unclear how important these differences are in practice as currently available comparisons of the various MI models in the literature are limited to a few methods and are in very specific settings [19, 21–26] and no comprehensive comparison of the available methods has been conducted.

In the current paper we present a comprehensive simulation-based comparison of the MI methods available in standard software packages for imputation of incomplete longitudinal data. Specifically, we evaluated estimators of regression coefficients for both a linear regression model and a linear mixed-effects model (LMM) in the presence of incomplete binary and continuous predictors. Our primary aim was to investigate whether the GLMM-based MI approaches, which are specifically designed for imputing longitudinal data, provide more accurate estimates than the standard MI approaches. We based our simulation study on a previously conducted analysis exploring the association between the burden of overweight and quality of life (QoL) in the Longitudinal Study of Australian Children (LSAC) [27].

## Methods

### Longitudinal study of Australian children (LSAC) data and analysis models

LSAC is a nationally representative study that examines the development and wellbeing of Australian children. Following recruitment, data have been collected every 2 years (referred to as waves of data collection) using face-to-face interviews, questionnaires and direct anthropometric measurements. The study is ongoing with six waves of data currently available. The detailed study procedure has been described elsewhere [28]. The simulation study was based on data from the kindergarten (K) cohort of LSAC (*n* = 4983), who were aged 4–5 years when recruited in 2004. The specific question of interest that we focused on was motivated by previous research examining whether the number of overweight occasions in waves 1–5 of data collection predicts poor QoL at wave 6, adjusted for child age, sex, English speaking background and family socio-economic position [27]. In this analysis, the child’s age-and-gender specific body mass index (BMI z-score) at each wave was dichotomized into normal weight (including underweight) and overweight (including obesity) using International Obesity Task Force criteria [29].

In our study we slightly modified the original analysis model to include an additional known predictor of child QoL: family structure (which also had missing data), reflecting whether the child was living with a single parent or two parents [30]. Specifically, we were interested in exploring:

1. the association between the cumulative burden of overweight from 4 to 5 to 12–13 years (waves 1–5) and QoL z-scores at age 14–15 years (wave 6), and
2. the cross-sectional association between QoL z-score and BMI z-score across all 6 waves,

as these two analysis models are of interest to many researchers in longitudinal data settings. Both analyses were adjusted for sex, English language background and socio-economic position (all at baseline), age and family structure.

The association between QoL z-score at wave 6 and cumulative burden of overweight (OverWtCat) were adjusted for child sex (Sex), English language background (language) and socio-economic position (SEP), all at baseline, age at wave 6 and burden of family structure(FamStCat). OverWtCat and FamStCat both are derived variables representing cumulative burden of overweight and living with a single parent, respectively. These variables were derived by counting the total number of waves, from 1 to 5, on which the child was overweight or lived with a single parent (followed by categorization as none, 1, 2, 3–4, or all five waves), respectively. More precisely, the analysis model to address (1) was a linear regression model:

1 $$ {\displaystyle \begin{array}{l}{\mathrm{QoLz}}_{i6}=\\ {}{\beta}_0+{\beta}_{11}I\left({\mathrm{OverWtCat}}_i=1\right)+{\beta}_{12}I\left({\mathrm{OverWtCat}}_i=2\right)+{\beta}_{13}I\left({\mathrm{OverWtCat}}_i=3\right)+{\beta}_{14}I\left({\mathrm{OverWtCat}}_i=4\right)+\\ {}{\beta}_2{\mathrm{ageyr}}_{6i}+{\beta}_3{\mathrm{Sex}}_{i1}+{\beta}_4{\mathrm{Sex}}_{i1}+{\beta}_5{\mathrm{language}}_{i1}+{\beta}_{61}I\left({\mathrm{FamStCat}}_i=1\right)+\\ {}{\beta}_{62}I\left({\mathrm{FamStCat}}_i=2\right)+{\beta}_{63}I\left({\mathrm{FamStCat}}_i=3\right)+{\beta}_{64}I\left({\mathrm{FamStCat}}_i=4\right)+{\varepsilon}_i,\\ {}\kern0ex \end{array}} $$

where *i* = 1, 2, …, *n* indexes the participant, QoLz_6_ is the QoL z-score at wave 6; ageyr_6_ represents the age (year) of the study child at wave 6; Sex, SEP and language represent sex, socio-economic position and English language, respectively measured at wave 1. The residual error *ε*_*i*_ was assumed to be normally distributed.

The analysis model to address (2) was a LMM applied to all 6 waves of data:

2 $$ {\mathrm{QoLz}}_{ij}={\gamma}_0+{\gamma}_1{\mathrm{BMIz}}_{ij}+{\gamma}_2{\mathrm{ageyr}}_{ij}+{\gamma}_3{\mathrm{Sex}}_{i1}+{\gamma}_4{\mathrm{SEP}}_{i1}+{\gamma}_5{\mathrm{language}}_{i1}+{\gamma}_6{\mathrm{FamStruc}}_{ij}+{u}_{0i}+{e}_{ij}, $$

where *i* = 1, 2, …, *n* and *j* = 1, 2, …, 6 index participants and waves respectively, BMIz is the BMI z-score, FamStruc represents whether the child was living with a single parent or two parents and *u*_0*i*_ represents a subject-specific random intercept. The other variables have the same definition as above. Note that in analysis model (1) only the QoL z-score data from wave 6 is analysed whereas analysis model (2) uses all the QoL z-score data.

Both analysis models were susceptible to missing data when applied to LSAC. Data were missing for BMI z-score and QoL z-score in all six waves. Family structure was completely observed for the first wave but had missing values in the subsequent waves. Age and sex were fully observed at baseline, but the other baseline variables had very small amounts of missing information (SEP 0.2% and English-speaking background, 1.4%). Age was occasionally missing at the later waves but was completed by adding the time difference between the wave and wave 1 to the child’s age at wave 1. To simplify our example, we excluded cases where QoL z-score was missing across all 6 waves (given that is the outcome of interest) and participants with missing socio-economic position, and/or English language background information at wave 1, leaving a total of 4661 participants for analysis. Except for QoL z-score, most of the missingness observed in our case study was due to dropout (Additional file 1: Table S1).

### MI methods to impute longitudinal data

We have identified the following MI methods available for imputing longitudinal data in standard software packages (see Table 1 for additional details).

1. JM-MVN: Originally implemented in Schafer’s NORM software [12], this approach utilizes data augmentation, a form of Markov chain Monte Carlo algorithm, to impute missing data under the assumption of (unstructured) multivariate normality. For longitudinal data, this approach treats repeated measurements as distinct variables and imputes all variables in the imputation model as continuous. The model is clearly mis-specified for imputing binary and categorical data, but JM-MVN has been shown to perform reasonably well even for such variables, unless they are severely skewed [12, 23, 31].
2. JM-MLMM: Instead of treating repeated measurements as distinct variables, Schafer and Yucel (2002) suggested using a joint multivariate LMM (JM-MLMM) for imputing several incomplete longitudinal variables [14]. This approach assumes all the incomplete variables are continuous with subject-specific random effects to incorporate dependence within individuals across time. As with the univariate LMM, this approach assumes that random effects and measurement errors follow a normal distribution with constant error-covariance for all individuals.
3. JM-MLMM-LN: Goldstein, Carpenter, Kenward and Levin. (2009) extended the JM-MLMM approach to impute a mixture of discrete and continuous variables [15], by treating  discrete variables to underlying latent normal (LN) variables that follow a multivariate normal distribution jointly with the continuous variables. The latent variable formulation is attractive because it offers computational advantages by modelling categorical variables within the established Bayesian estimation procedure for normally distributed variables and transforms between the latent variable and the original binary/categorical variables using a probit model to provide imputed values on the categorical scale.
4. FCS-Standard: The standard implementation of FCS includes all the repeated measurements of the time-varying covariate as predictors in each of the univariate imputation models; we refer to this approach as FCS-Standard. This method is subject to convergence problems due to model over-fitting and/or collinearity when there are a large number of correlated repeated measures.
5. FCS-Twofold: To overcome convergence problems with FCS-Standard when applied to longitudinal data, Nevalainen et al. (2009) proposed the FCS-Twofold method in which the imputation model for incomplete time-dependent variables only includes measurements within pre-specified time blocks (21). This approach incorporates nested iterations that cycle both within and between time blocks. The requirement for multiple iterations in each time block makes FCS-Twofold computationally demanding.
6. FCS-MTW: An alternative approach to FCS-Twofold is to apply the same restriction ("moving time window", MTW) to the time-dependent covariates in the univariate models but remove the within-time iterations from the algorithm.
7. FCS-LMM: Instead of treating repeated measurements as distinct variables, the FCS-LMM method uses a multilevel LMM for imputing missing values in each incomplete time-dependent variable given all the others, cycling iteratively through the univariate imputation models. This method implements the Gibbs sampler for the linear two-level model with homogeneous within-subject variances, which is a special case of a multivariate LMM, using the same imputation models as JM-MLMM but with only one variable considered missing at each iteration.
8. FCS-LMM-het: Van Buuren (2011) extended FCS-LMM to allow for subject-specific error variances/heteroscedastic variance [19]. As with FCS-LMM, in this approach the binary and categorical variables are imputed as continuous variables.
9. FCS-GLMM: Resche-Rigon and White (2016) proposed an extension of FCS-LMM that imputes both continuous and binary incomplete variables using appropriate GLMMs (18). This method uses identity and logit links to impute missing data in continuous and binary variables, respectively.
10. FCS-MLMM-LN: Audigier and Resche-Rigon suggested that the JM-MLMM-LN approach could be modified to impute missing data in an FCS framework where only one variable is considered missing at a time [33]. At each step, this method treats all the variables in the imputation model (one incomplete and the rest as complete variables) as outcomes (hence the MLMM nomenclature) and integrates the likelihood over the missing responses of the incomplete variable to obtain the observed data likelihood. Within this specification binary and categorical incomplete variables are imputed using latent normal variables as for JM-MLMM-LN.
11. FCS-LMM-LN: Enders, Keller and Levy (2017) proposed extensions of both FCS-LMM and FCS-LMM-het that uses a latent normal variable for imputing incomplete categorical variables [18].
12. FCS-LMM-PMM: In this final extension of GLMM-based FCS approaches, missing values are imputed by drawing an observation randomly from a set of complete cases (donors) having predicted means close to that of the incomplete mean. The match is based on predicted values from a linear mixed effects imputation model that contains both fixed and random effects.

**Table 1:** Summary of imputation approaches for handling missing data in longitudinal studies available in standard software

MI approaches	Method	Details	Software
Joint modelling (JM) (Assumes a joint multivariate distribution between all the variables in the imputation model)	JM-MVN	• Repeated measurements of time-dependent variables are imputed as distinct variables. • Assumes a joint multivariate normal distribution for all incomplete variables. • Binary variables are imputed as continuous variables. • Categorical variables can be imputed as a continuous variable or as a series of dummy variables.	SAS (7), SPSS (42), Stata (8), Mplus (43) and R (9)
	JM-MLMM	• Repeated measurements of time-dependent variables are imputed using hierarchical models. • All incomplete variables are imputed using a joint multivariate LMM. • Binary variables are imputed as continuous variables. • Categorical variables can be imputed as a continuous variable or as a series of dummy variables. • A constant residual error variance is assumed for all individuals.	Mplus, R package pan [42].
	JM-MLMM-LN	• Repeated measurements of time-dependent variables are imputed using hierarchical models. • All incomplete variables are imputed using a joint multivariate LMM. • Binary and categorical incomplete variables are imputed using latent normal variables. • Can be fitted assuming either a constant or a subject-specific residual error variance.	Realcom-impute [43], R package jomo [16].
Fully conditional specification (FCS) (Imputes using a univariate conditional model for each variable with missing data)	FCS-Standard	• Repeated measurements of time-dependent variables are imputed as distinct variables. • Imputes variables using conditional univariate regression models for each incomplete variable, conditional on the time-dependent variables at all waves.	SAS, SPSS, Stata, Mplus and R
	FCS - Twofold	• Repeated measurements of time-dependent variables are imputed as distinct variables. • Imputes variables using univariate regression model for each incomplete variable, conditional on a subset of all time-dependent variables in the data based on a window period. • Imputation carried out in a two-step iterative process.	Stata
	FCS-MTW	• Repeated measurements of time-dependent variables are imputed as distinct variables. • Imputes variables using univariate regression models for each incomplete variable, conditional on a subset of all time-dependent variables in the data based on a window period. • Imputation carried out in a single step iterative process.	Stata
	FCS-LMM	• Repeated measurements of time-dependent variables are imputed using hierarchical models. • Assumes a conditional LMM for each incomplete variable. • Binary variables are imputed as continuous variables. • Categorical variables can be imputed as a continuous variable or as a series of dummy variables. • A constant residual error variance is assumed for all individuals.	R package mice (*mice.impute.2 l.pan)* [44].
	FCS-LMM-het	• Repeated measurements of time-dependent variables are imputed using hierarchical models. • Assumes a conditional LMM for each incomplete variable. • Binary and categorical variables are imputed as continuous variables. • The model assumes a subject-specific residual error variance.	R package mice (*mice.impute.2 l.norm)* [44].
	FCS-GLMM	• Repeated measurements of time-dependent variables are imputed using hierarchical models. • Assumes a conditional GLMM for incomplete binary and categorical variables. • A constant residual error variance is assumed for all individuals	R package micemd [33]
	FCS-MLMM-LN	• Repeated measurements of time-dependent variables are imputed using hierarchical models. • Only a single variable is considered to be missing in a given iteration and is imputed using a joint LMM similar to JM-MLMM-LN using imputed values for the other incomplete variables. This process is repeated for all incomplete variables in turn. • Binary and categorical incomplete variables are imputed using a latent normal variable. • Can be fitted using either a constant or a subject-specific residual error variance.	Mplus, R package micemd
	FCS- LMM-LN	• Repeated measurements of time-dependent variables are imputed using hierarchical models. • Assumes a conditional LMM for incomplete variables. • Binary and categorical incomplete variables are imputed using a latent normal variable • Can be fitted using either a constant or a subject-specific residual error variance.	Blimp [45]
	FCS-LMM-PMM	• Repeated measurements of time-dependent variables are imputed using hierarchical models. • Imputes incomplete values using a draw from a pool of observed values who have the closest predicted mean to that of the incomplete case.	R package miceadds [46]

The following abbreviations are used to denote different MI methods, e.g., MVN: multivariate normal imputation; MLMM: Multivariate linear mixed-effects model; MLMM-LN: Multivariate linear mixed-effects model with latent normal variables; LMM: Linear mixed-effects model; PMM-Predicted mean matching; GLMM-Generalised linear mixed-effects model; MTW – Moving Time Window

Although some of the above methods (JM-MLMM-LN, FCS-LMM-het, FCS-MLMM-LN and FCS-LMM-LN) can allow the subject-specific error variance to vary across individuals, doing so may not produce stable results in the case of LMM with only random intercepts. We therefore considered homoscedastic error variance for all the methods where possible (all but FCS-LMM-het).

### Simulation study

#### Data simulation

To compare the behaviour of the regression coefficient estimators using the above MI approaches, we generated 1000 datasets, each with eight variables (age and sex of the child, English language background, mother’s highest education level (whether completed year 12), family structure, socio-economic position of the family, child’s BMI z-scores and QoL z scores) and 5000 individuals. In each dataset, variables were simulated in a sequential manner as follows:

1. Child sex, whether English is the main language spoken at home (language) and mother’s education (moedu*:* whether or not completed year 12) at baseline were generated using binomial distributions with probabilities 0.5, 0.9 and 0.6 respectively.
2. Child age (in years) at each wave was generated according to the following model:

3 $$ {\mathrm{ageyr}}_{i\mathrm{j}}=\frac{1}{12}\left\{48+\left({\mathrm{wave}}_{\mathrm{j}}-1\right)\ast 24+{u}_{i.}+{v}_{ij}\right\} $$

where 48 + *u*_*i*._ = 48 + *N*(11,1.5), is the distribution of age (in months) of the participant at the recruitment and *v*_*ij*_ = *N*(0, 2), j = 2,3,...,6, is the random noise in the age distribution to accommodate the random variation of the age at interview during follow-up as observed in the LSAC.

3. The family structure variable (FamStruc, whether the child was living with a single parent or two parents) in each wave was simulated using the following logistic mixed-effects model:

4 $$ \mathrm{logit}\left(P\left({\mathrm{FamStruc}}_{ij}=1\right)\right)=-6.9+0.15\ast {\mathrm{ageyr}}_{ij}+0.62\ast {\mathrm{language}}_i-1.9\ast {moedu}_i+{\xi}_{0i} $$

where *ξ*_*oi*_ = *N*(0,5.5) is the random intercept.

4. The socio-economic position (SEP) at baseline was generated based on mother education and family structure at baseline (wave = 1) using the following model:

5 $$ {\mathrm{SEP}}_{i1}=-0.5+0.94\ast {\mathrm{moedu}}_i-0.45\ast {\mathrm{FamStruc}}_{i1}+{\tau}_i $$

where, *τ*_*i*_ = *N*(0,1.2) is the residual error.

5. The main exposure variable of interest, child BMI z-score was generated based on age, sex and family structure using the following linear mixed-effects model:

6 $$ {\mathrm{BMIz}}_{ij}=0.53+\left(-0.012\right)\ast {\mathrm{ageyr}}_{ij}+\left(-0.008\right)\ast {\mathrm{sex}}_{i1}+0.07\ast {\mathrm{FamStruc}}_{ij}+{\varphi}_{oi}+{\varphi}_{ij} $$

where *φ*_*oi*_ = *N*(0,0.9) is the random intercept and *φ*_*ij*_ = *N* (0,0.6) is the residual error.

6. Finally, the outcome, QoL z-score at each wave was generated using the following linear mixed-effects model:

7 $$ {\mathrm{QoLz}}_{ij}=-0.04+0.05\ast {\mathrm{sex}}_{i1}-0.02\ast {\mathrm{ageyr}}_{ij}+\left(-0.12\right)\ast {\mathrm{BMIz}}_{ij}+\left(-0.2\right)\ast {\mathrm{FamStruc}}_{ij}+0.20\ast {\mathrm{language}}_{i1}+0.09\ast {\mathrm{SEP}}_{i1}+{\omega}_{0i}+{\omega}_{ij} $$

where *ω*_0*i*_~*N*(0,0.7) and *ω*_*ij*_~*N*(0,0.7).

Note that we used a single linear mixed model (7) to generate data for both analysis models (1) and (2). Thus, for analysis model (2), the regression coefficients used in the data generating model were considered as the true values. The true values of the regression coefficients for analysis model (1) were estimated by generating a simulated population of 10 million individuals and fitting the model of interest. All the parameters in the above data generating models were estimated from LSAC data to ensure that the simulated datasets were comparable to a real data example.

As both analysis models were susceptible to missing data when applied to LSAC, upon generating the complete datasets, we set BMI z-scores for all waves and family structure from waves 2 to 6 to missing according to the MAR assumption. Specifically, we used the following equation to induce missingness:

i. Values of BMI z-score at *j*^*th*^ wave (j = 1, 2,…,6) were set to missing dependent on QoL z-score and age at that wave:

8 $$ logit\left(P\left({\mathrm{BMIz}}_{ij}=\mathrm{missing}\right)\right)={\theta}_{0j}+{\theta}_{1j}{\mathrm{ageyr}}_{ij}+{\theta}_{2j}{\mathrm{QoLz}}_{ij} $$

ii. Similarly, family structure in waves 2 to 6 was set to missing at a given wave dependent on baseline family structure and the value of the child QoL z-score of that wave:

9 $$ logit\left(P\left({\mathrm{FamStruc}}_{ij}=\mathrm{missing}\right)\right)={\vartheta}_{0j}+{\vartheta}_{1j}{\mathrm{FamStruc}}_{i1}+{\vartheta}_{2j}{\mathrm{QoL}z}_{ij} $$

The parameters for the above model (*θ*_0_, *θ*_1*j*_, *θ*_2*j*_ and *ϑ*_0_, *ϑ*_1*j*_, *ϑ*_2*j*_) were chosen based on the LSAC data to ensure a similar proportion of missing observations for each of the variables at each wave as in LSAC. A summary of the proportions of missing data by wave in both the LSAC and simulated data is given in Table 2.

**Table 2:** Comparisons of the missing data proportions in both LSAC and simulated data

Data collection wave	Proportion of missing data in BMI z-score		Proportion of missing data in FamStruc	
	Case study	Simulation study	Case study	Simulation study
1	0.06	0.01	0.00	0.00
2	0.08	0.06	0.03	0.06
3	0.09	0.10	0.05	0.08
4	0.14	0.15	0.09	0.10
5	0.19	0.19	0.14	0.15
6	0.30	0.28	0.24	0.24

We have also provided parameter values for the above models in the Additional file 1: Table S2, see supplementary materials. Although in the LSAC data and in many longitudinal studies the outcome variable might also have missing values, in our simulation study, we considered the outcome to be fully observed, in order to ensure that the simulated missing data scenarios satisfied the MAR assumption.

### Imputation strategies and comparison of methods

We applied each of the 12 imputation models described above to both the simulated and LSAC datasets. For each MI approach, we imputed continuous BMI z-score, binary family structure variable and QoL z-score (only in the case study) at each wave. The imputation model also included age and sex of the child, socio-economic position and whether English was the main language spoken at home at Wave 1, and QoL z-score at each of the 6 waves. For MI methods that impute binary variables as continuous in the imputation model, we rounded the imputed values (to either 0 or 1) using adaptive rounding [31] for analysis model (1), as rounding was required to derive the FamStCat variable (i.e., the number of waves that the participant lived with a single parent). However, for analysis model (2) we used the unrounded values in the analysis as rounding can introduce bias [11, 34]. Forty imputations were generated for each approach to limit Monte Carlo (imputation-related) error for the regression coefficient of interest to approximately 5% of its standard error. Of note, for both FCS-MTW and FCS-Twofold method we used measurements within adjacent time-period in the imputation model.

Using simulated datasets, we compared the bias, standard errors (both average of the model based and empirical standard error) and coverage probability of the estimated regression coefficients among the 12 imputation approaches, complete data analysis (fully observed simulated dataset of 5000 observations, before inducing missingness) and available data analysis (that excludes patients/records with missing data). The sampling properties of the estimators are estimated from 1000 simulated datasets.

## Results

### Simulation results

**Fig. 1:**
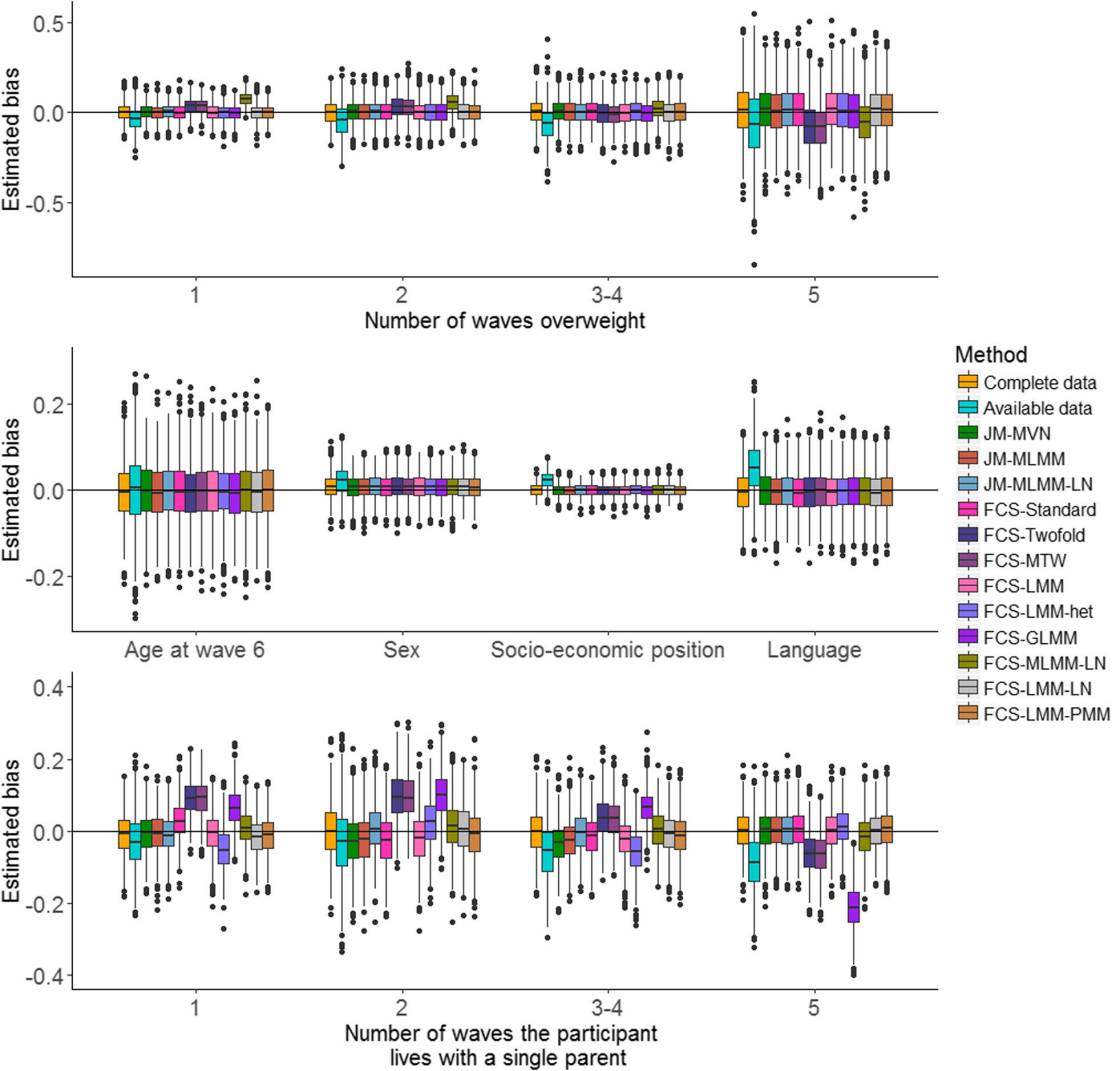
Distribution of the bias in the estimated regression coefficients (i.e., mean changes in the QoL z-score associated with each covariate) for analysis model (1) across the 1000 simulated datasets following complete data, available data and 12 multiple imputation methods. Top and bottom panel show the distribution of the bias in the estimated regression coefficients for covariates with missing data whereas the middle panel shows the distribution of the bias associated with fully observed covariate

**Fig. 2:**
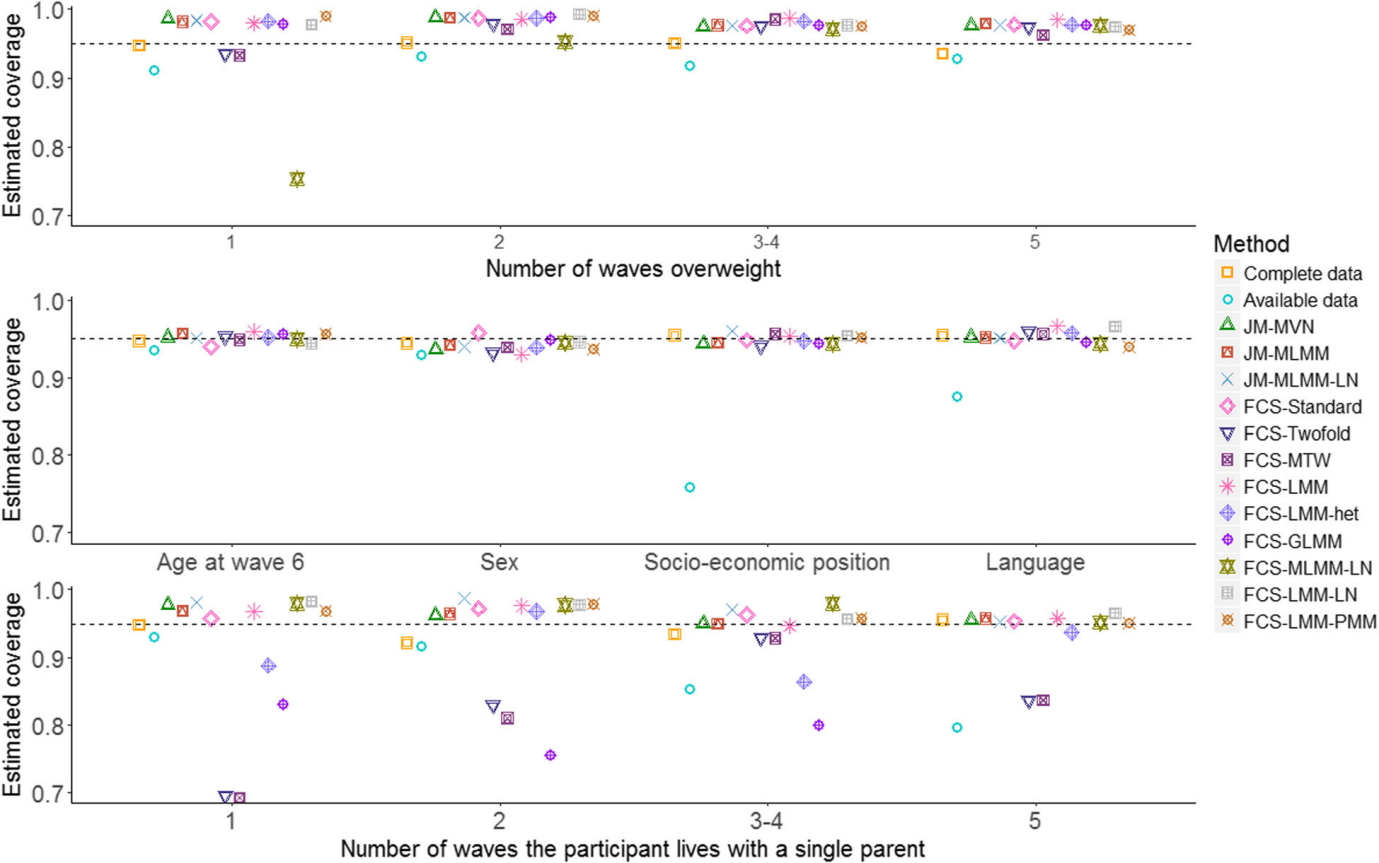
Estimated coverage of the 95% confidence interval for the regression coefficients in analysis model (1), derived from 1000 simulated datasets. The dotted lines indicate the nominal value of 95%

The sampling distribution of the estimated bias and the coverage of the regression coefficients for analysis model (1) across the 1000 simulated datasets are displayed in Figs. 1 and 2, respectively. A detailed numerical summary of the estimated bias and standard errors is provided in Additional file 1: Figure S1. It is clear from Figs. 1 and 2 that analysis based on the available data resulted in biased estimation of the regression coefficients, and inadequate coverage probabilities. All the MI approaches provided similar estimation of the regression coefficients for fully observed covariates (age at wave 6, sex, socio-economic position and language). The estimated coverage probabilities for these covariates were very close to the nominal value of 0.95. Slightly greater differences were observed across the imputation methods for regression coefficients associated with incomplete predictors. The estimated bias in the regression coefficients for the cumulative burden of overweight was similar across all the methods except perhaps FCS-Twofold, FCS-MTW and FCS-MLMM-LN. For the cumulative burden of living with single parents all methods gave similar estimates of the regression coefficients except FCS-Twofold, FCS-MTW, and FCS-GLMM, which also exhibited some under-coverage.

**Fig. 3:**
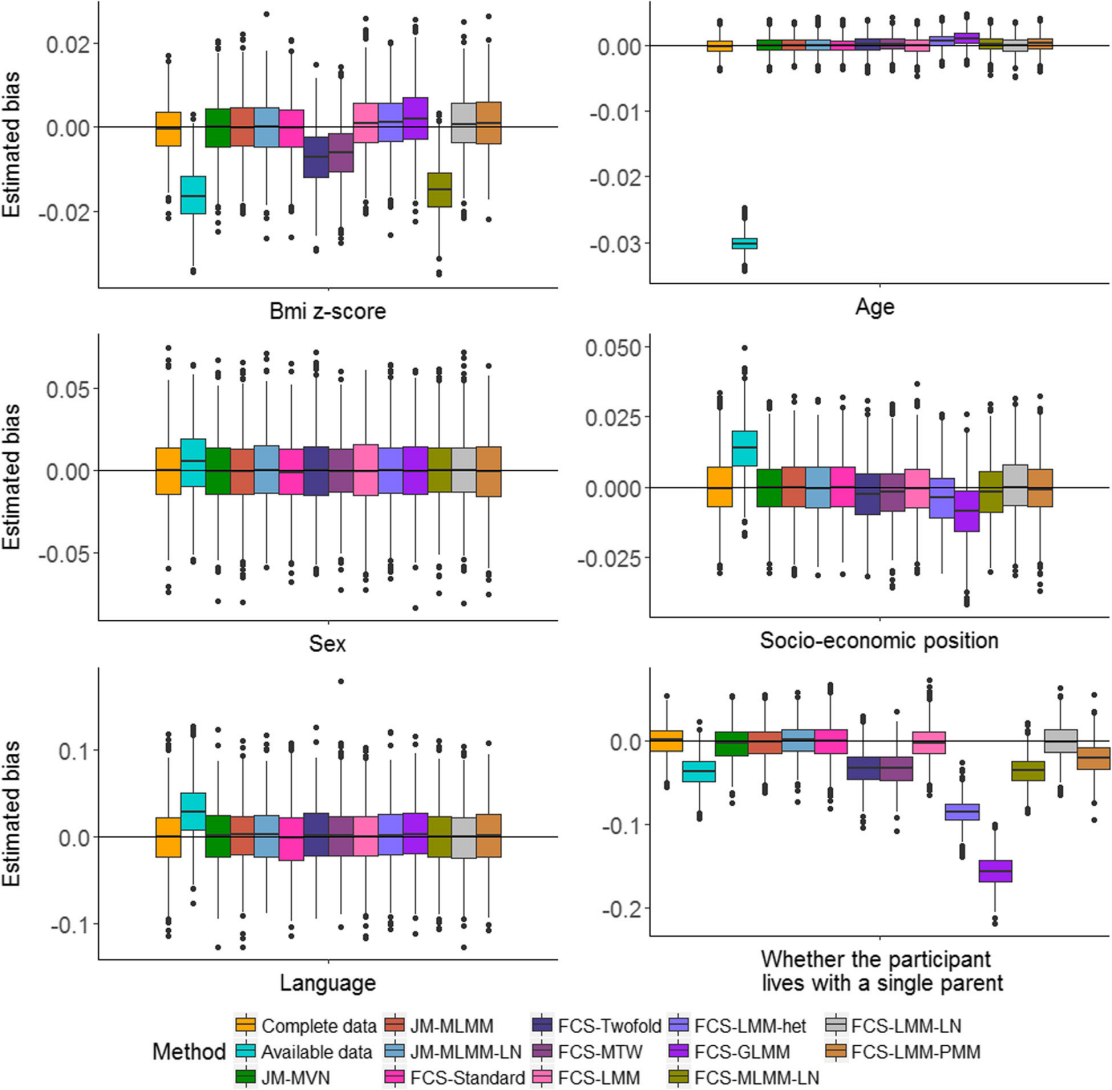
Distribution of the bias in the estimated regression coefficients (i.e., mean changes in the QoL z-score associated with each covariate) for analysis model (2) across the 1000 simulated datasets following complete data, available data and 12 multiple imputation methods. Top, left and bottom right panels show the distribution of the bias in the estimated regression coefficients for covariates with missing data and all other panels show the distribution of the bias associated with fully observed covariate

**Fig. 4:**
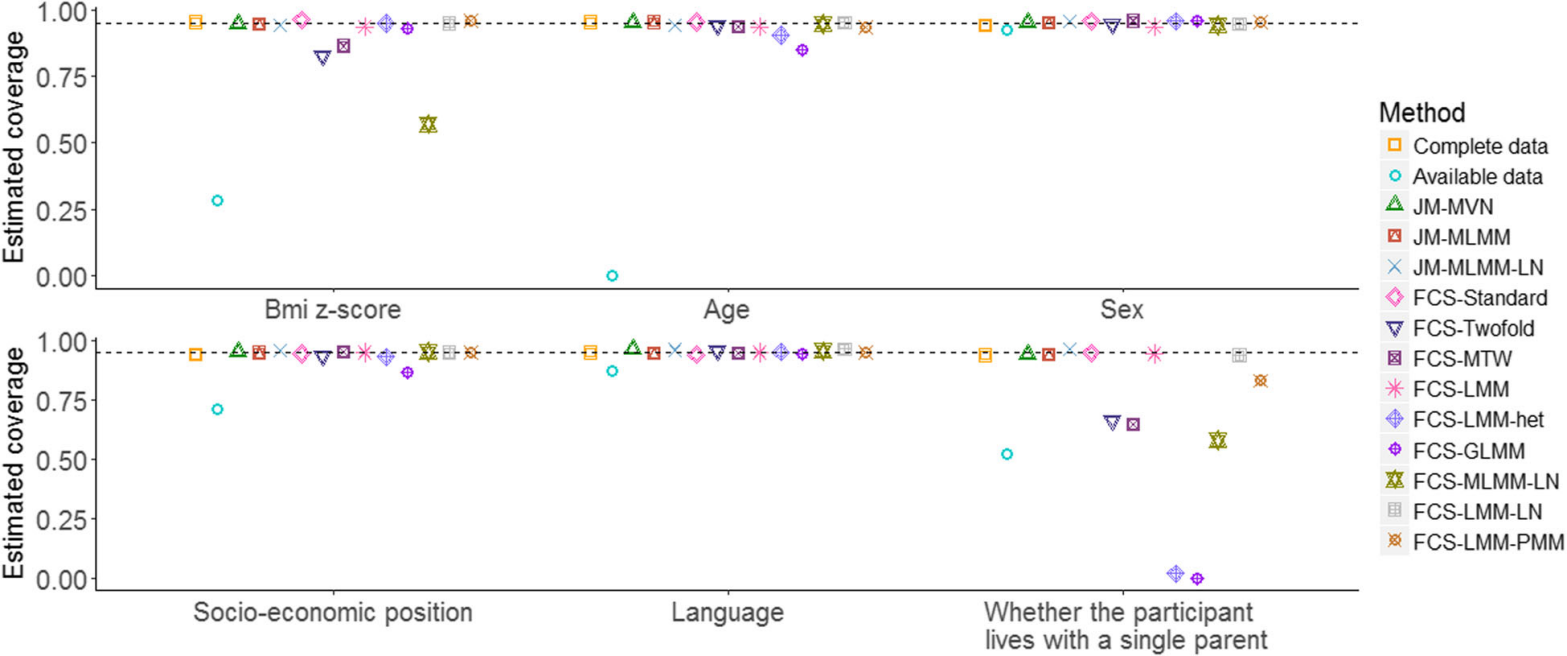
Estimated coverage of the 95% confidence interval for the regression coefficients in analysis model (2), derived from 1000 simulated datasets. The dotted lines indicate the nominal value of 95%

For analysis model 2 (the LMM), we observed mixed results across MI methods in performance associated with incomplete predictors (Figs. 3 and 4). Again, the available data analysis, FCS-Twofold, FCS-MTW and FCS-MLMM-LN resulted in biased estimates of the regression coefficients and under-coverage for both the incomplete predictors. In addition, FCS-GLMM, FCS-LMM-het and FCS-LMM-PMM resulted in biased estimation of regression coefficients and under-coverage for the family structure indicator. All of the MI approaches demonstrated reliable estimation of the intra-cluster correlation (Additional file 1: Figure S1).

**Fig. 5:**
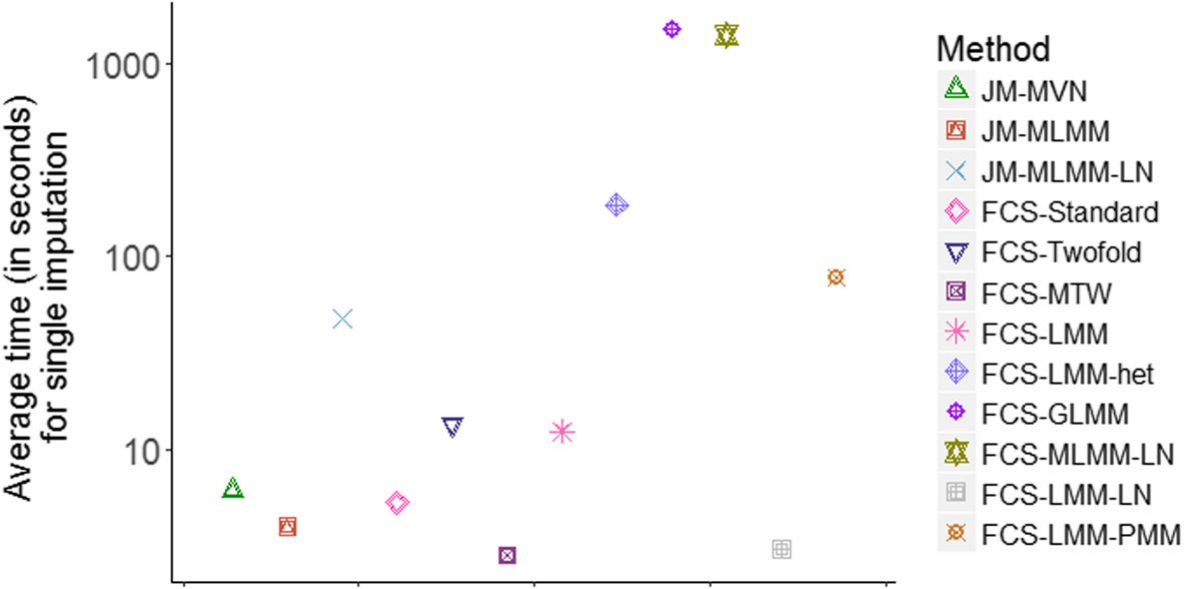
Average computational time (in seconds) for single imputation for each of the MI methods when applied to a single simulated dataset

The implementation time of all of the 12 methods (implemented on a standard Windows PC intel core i-5, 3.2 GHz processor and 8GB RAM) is summarized in Fig. 5. Briefly, FCS-Standard, JM-MVN, JM-MLMM, FCS-Twofold, FCS-MTW, FCS-LMM and FCS-LMM-LN required around 10 s for single imputation when applied to the simulated data, with FCS-MTW and FCS-LMM-LN taking the least computational time. Both the standard cross-sectional approaches (JM-MVN and FCS-Standard) required similar amounts of computational time. On the contrary, JM-MLMM-LN, FCS-LMM-PMM, FCS-LMM-het, FCS-GLMM and FCS-MLMM-LN were more computationally expensive requiring approximately 10 (for JM-MLMM-LN, FCS-LMM-PMM, FCS-LMM-het) to 100 (FCS-GLMM and FCS-MLMM-LN) times longer implementation time compared with the standard cross-sectional approaches. The latter two methods may not be feasible in practice because of these long computational times.

### Analysis of LSAC data

**Fig. 6:**
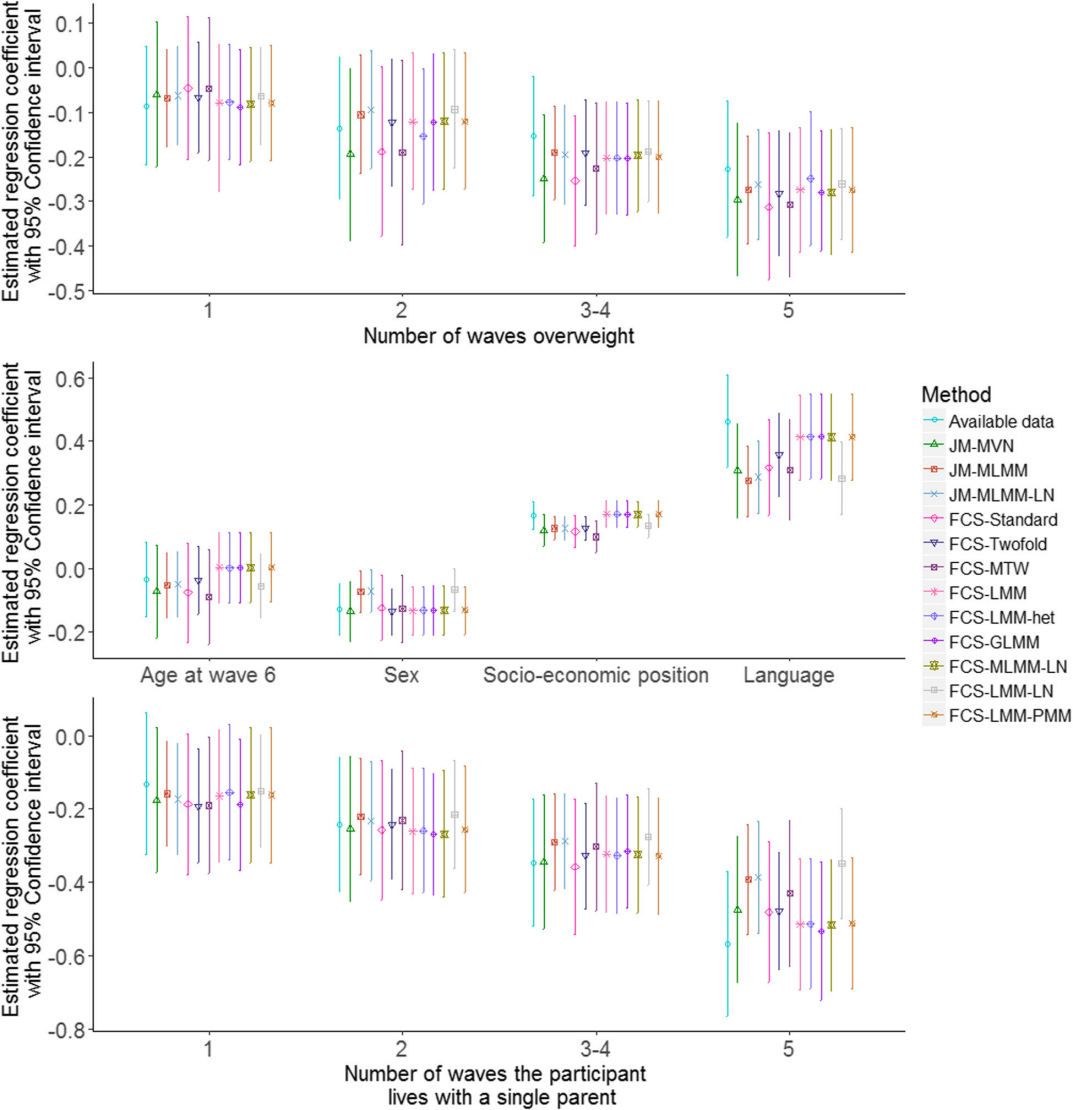
Estimated regression coefficients and 95% CI for analysis model (1) applying available data and all the approaches to handle missing data in LSAC

**Fig. 7:**
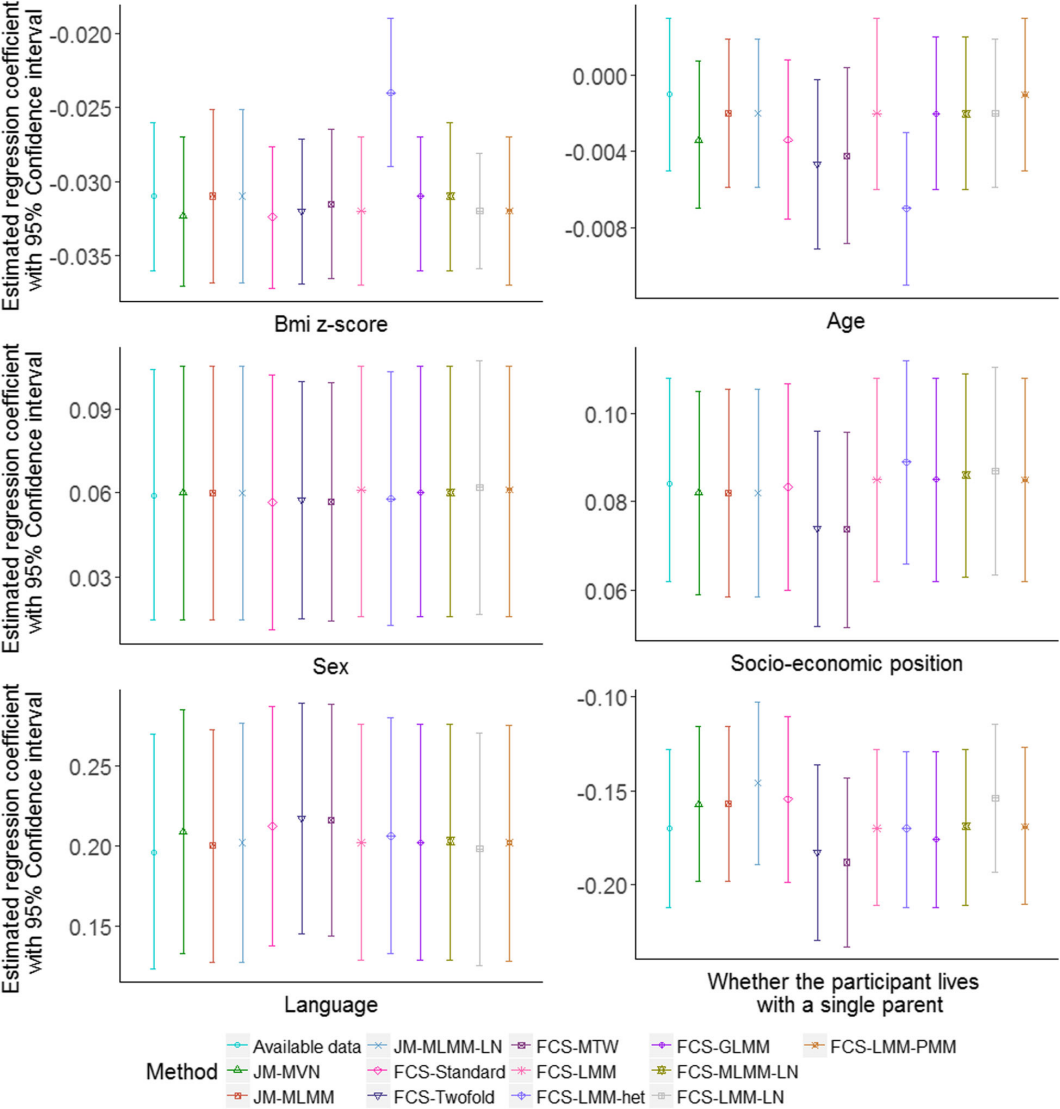
Estimated regression coefficients with 95% CI for analysis model (2) applying available data and all the MI approaches to handle missing data in LSAC

Estimated regression coefficients with 95% confidence intervals (CIs) for the two analysis models applied using different imputation approaches to the analysis of the LSAC data are given in Figs. 6 and 7. Both the figures suggest an inverse association between BMI and QoL. For both analysis models, we observed that FCS-Standard and JM-MVN resulted in very similar estimates of the regression coefficients and 95% CIs. The estimated regression coefficients and corresponding 95% CIs for other methods were also generally similar, although some variability among the approaches was observed, especially in some of the results for FCS-LMM-LN and FCS-LMM-het for analysis models (1) and (2), respectively.

## Discussion

In this paper we evaluated the performance of currently available MI methods for handling incomplete variables in longitudinal studies in the context of estimating regression coefficients from a linear regression model (with a cumulative measure of exposure) and a LMM, two commonly used models in the analysis of longitudinal data. We found that FCS-Standard and JM-MVN provided reliable estimates of the regression coefficients for both analysis models, often with better coverage probabilities than the GLMM-based methods (FCS-LMM-het, FCS-GLMM, FCS-MLMM-LN, and FCS-LMM-PMM). JM-MLMM and FCS-LMM, although mis-specified for imputing binary variables, resulted in reliable estimation of the regression coefficients for both analysis models. In addition, JM-MLMM-LN and FCS-LMM-LN also exhibited great promise.

The FCS-Twofold and FCS-MTW methods produced slightly more biased and less precise estimates than FCS-Standard. The observed bias was likely to be because these approaches limit the variables in the univariate imputation models, thereby potentially throwing away important information about the missing data. Similar bias in the estimated regression coefficients following FCS-Twofold was observed by De Silva and colleagues [22], who showed that this bias can be reduced by relaxing the restriction to include a wider time window. In our simulations, we observed very similar results from FCS-Twofold and FCS-MTW, casting doubt on the need for the nested within- and among-time-point iterations in the FCS-Twofold approach.

We observed relatively poor performance for several GLMM-based approaches, namely FCS-LMM-het, FCS-GLMM, FCS-MLMM-LN and FCS-LMM-PMM. Bias in the estimated regression coefficients for binary covariates following FCS-LMM-het was also reported in a previous simulation study [35]. The poor-performance of FCS-MLMM-LN might be due to its implementation in the FCS framework which requires many iterations to converge. Moreover, this method is computationally demanding, limiting its usefulness in practice. Finally, FCS-LMM-PMM appeared to perform relatively poorly in our simulation study, especially for binary incomplete variables. It would be important to explore the use of this approach in further datasets as it has been shown that predictive mean matching provides good results in the context of cross-sectional data [36].

In general, our results suggest that using either FCS-Standard or JM-MVN produces reliable estimates of the regression coefficients for incomplete longitudinal data. These methods are known to be equivalent in the case of normally distributed covariates [23, 37], but for incomplete data in both binary and continuous covariates, the FCS-Standard approach can be more robust than a mis-specified joint modelling approach [38]. This is because for the joint incomplete binary and continuous data the FCS-Standard approach produces imputed values that are compatible with a restricted general location joint model. But for many situations, FCS-Standard may not correspond to a joint model when the conditional models are mis-specified, potentially resulting in sub-optimal imputation [39]. A detailed study of compatibility in the context of longitudinal data is beyond the scope of the present paper but warrants future study.

Both the JM-MVN and FCS-Standard approaches require study designs with a fixed number of sampling waves and fixed time intervals between successive waves. Therefore, these methods may not be appropriate if data are collected in irregular time intervals, and GLMM based approaches should be used. We found that several GLMM-based methods (such as JM-MLMM, JM-MLMM-LN, FCS-LMM, and FCS-LMM-LN) are potentially useful alternatives. In our simulation study we observed that although the JM-MLMM approaches misspecify the binary variable, it produces reliable estimates of the regression parameters. This method uses the inverse Wishart prior for modelling the covariance matrices, hence avoiding the convergence issues of the Gibbs sampler. It also requires a relatively small amount of computational time. Comparable estimates of the regression parameters and coverage were obtained from JM-MLMM, JM-MLMM-LN and FCS-LMM. JM-MLMM-LN approach avoids misspecification of binary variables by specifying a latent normal distribution for binary variables, but this method is more computationally expensive than JM-MLMM. The suboptimal performance of the FCS-LMM-het approach compared with FCS-LMM might lie with the specification of residual error variance. The FCS-LMM method assumes a common residual variance for all individuals while FCS-LMM-het assumes subject-specific residual variances which may not be well estimated from longitudinal data with few repeated measurements. Moreover, the FCS-LMM-het approach requires all the variables in the imputation model to have both random intercepts and slopes, hence is computationally expensive and may suffer convergence problems. For this reason, its usefulness in the context of longitudinal data with binary variables is limited.

The results presented here are consistent with the results of a number of previous simulation studies. Comparable results between JM-MLMM and FCS-LMM were also obtained by Zhao and Yucel [32]. Our findings are also consistent with those of Kalaycioglu et al. (2013), who showed that JM-MLMM produced better results, both in terms of bias and precision, than FCS-MTW and FCS-LMM-het, with FCS-LMM-het performing the worst [35]. Similarly consistent results were reported by Audigier et al. (2018), who compared FCS-LMM-het, FCS-GLMM, JM-MLMM-LN methods for imputing incomplete binary and continuous data in the context of individual patient data meta-analysis, and found that JM-MLMM-LN performed better than FCS-LMM-het and FCS-GLMM [26]. Audigier et al. also considered an additional “FCS-2stage” method that fits separate imputation models within each cluster and then combines the results using a multivariate random effects model. They reported that FCS-2stage performed worse than FCS-GLMM and FCS-LMM-het when the cluster size was small. Given that in our study, and often in longitudinal studies, we only had a few waves of data collection, we did not include this approach in our comparison. Our results are also consistent with those of Enders et al. (2016) who showed that JM-MLMM-LN provided better results than FCS-LMM-het for imputing incomplete binary data in the context of a LMM with a random intercept [21].

Although our simulation study was based on a real dataset and thus has a realistic level of complexity, it is always difficult to draw general recommendations from a single simulation study. Further extensions could be to explore the behaviours of these MI methods when data are collected at irregular time intervals, when neither JM-MVN nor the FCS-Standard will be feasible. In our simulation study we only considered covariates to be missing and the outcome to be fully observed. This is because we restricted our simulation study to MAR scenarios, in which missingness in the covariate depends only on the outcome of interest and other (fully observed) covariates in the model. Although it may be reasonable to include a case where both outcome and covariates are missing this may introduce additional complexity with the data being missing not at random (MNAR). Exploring the sensitivity of the performance of the MI methods under various missingness assumptions is beyond the scope of the present paper. Both our simulations and case study are based on simplistic imputation models with few variables. However, in many situations the multiple imputation method may use high-dimensional data with a large number of predictors, in such situations JM-MVN and FCS-Standard may incur convergence problems. To overcome convergence issues, several methods, including using principal component analysis to reduce the dimensionality of the predictors in the imputation model, have been proposed [40, 41]. However, a full exploration of methods proposed to address the complexities arising in high-dimensional data, e.g., appropriate selection of predictors and convergence issues is beyond the scope of the present paper. Despite the use of a simplistic imputation model, this study has for the first time provided an overview and a systematic comparison of a growing number of approaches to MI with longitudinal data; as methods continue to develop, further evaluations will undoubtedly be needed.

## Conclusion

In summary, both the cross-sectional MI methods (FCS-Standard and JM-MVN) and some GLMM-based approaches (JM-MLMM, JM-MLMM-LN, FCS-LMM and FCS-LMM-LN) performed well for the estimation of regression parameters in the case of a linear regression model and LMM. Both approaches have important strengths and limitations. No single method is appropriate for every situation. As the GLMM-based approaches are still developing and are generally more complex than the cross-sectional methods, these may only be needed in specific circumstances such as irregularly spaced data or high-dimensional data that create convergence problems.

## Additional file

Additional file 1: **Table S1** and **S2** contains values of the average biases, average of the model standard errors and empirical standard errors under analysis model (1) and (2), respectively over 1000 simulated datasets. **Figure S1** shows the distribution of estimated intra-cluster correlation coefficients for analysis model (2). (DOCX 787 kb)

## Abbreviations

BMI: Body mass index
FCS: Fully conditional specification
GLMM: Generalized linear mixed-effect model
JM: Joint Modelling
LMM: Linear mixed-effect model
LN: Latent Normal
LSAC: Longitudinal Study of Australian Children
MAR: Missing at random
MI: Multiple imputation
MICE: Multiple imputation using chain equation
QoL: Quality of life
