# Supplementary material for: A comparison of multiple imputation methods for missing data in longitudinal studies
Source: BMC Med Res Methodol. 2018 Dec 12;18:168. doi: 10.1186/s12874-018-0615-6 (PMC6292063; doi:10.1186/s12874-018-0615-6)
Supplement: Supplementary file 1 — Table S1 and S2 contains values of the average biases, average of the model standard errors and empirical standard errors under analysis model (1) and (2), respectively over 1000 simulated datasets. Figure S1 shows the distribution of estimated intra-cluster correlation coefficients for analysis model (2). (DOCX 787 kb) [file 12874_2018_615_MOESM1_ESM.docx]

*
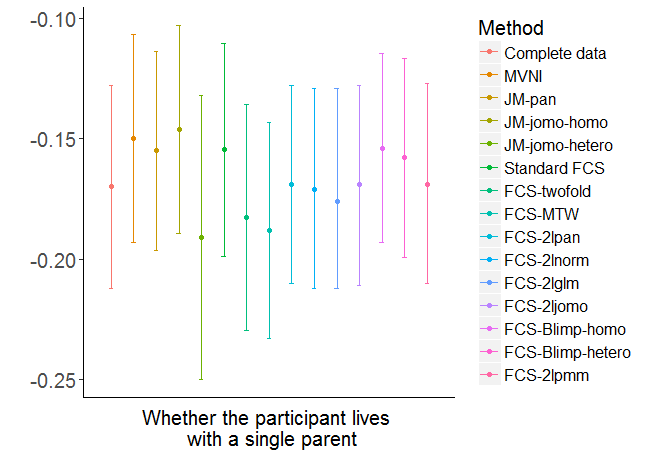
Table S1: Average bias and standard errors in the estimated regression parameters under analysis model (1).*

|  | Number of waves overweight | | | | | | | | | | | |
| --- | --- | --- | --- | --- | --- | --- | --- | --- | --- | --- | --- | --- |
| Methods | 1 | | | 2 | | | 3-4 | | | 5 | | |
|  | Bias^a^ | SE^b^ | SE^c^ | Bias^a^ | SE^b^ | SE^c^ | Bias^a^ | SE^b^ | SE^c^ | Bias^a^ | SE^b^ | SE^c^ |
| Complete data | 0.004 | 0.048 | 0.048 | 0.003 | 0.070 | 0.069 | 0.005 | 0.072 | 0.072 | 0.014 | 0.142 | 0.145 |
| Available data | -0.033 | 0.063 | 0.065 | -0.043 | 0.094 | 0.090 | -0.061 | 0.098 | 0.096 | -0.060 | 0.200 | 0.208 |
| JM-MVN | 0.003 | 0.053 | 0.044 | 0.007 | 0.079 | 0.060 | 0.006 | 0.078 | 0.066 | 0.017 | 0.152 | 0.131 |
| JM-MLMM | <0.001 | 0.053 | 0.043 | 0.005 | 0.079 | 0.062 | 0.003 | 0.078 | 0.065 | 0.010 | 0.152 | 0.131 |
| JM-MLMM-LN | 0.004 | 0.053 | 0.043 | 0.006 | 0.060 | 0.004 | 0.004 | 0.078 | 0.068 | 0.017 | 0.151 | 0.131 |
| FCS-Standard | <0.001 | 0.053 | 0.044 | 0.005 | 0.079 | 0.059 | 0.006 | 0.078 | 0.068 | 0.017 | 0.151 | 0.129 |
| FCS-Twofold | 0.037 | 0.053 | 0.042 | 0.032 | 0.078 | 0.061 | -0.005 | 0.079 | 0.069 | -0.072 | 0.164 | 0.139 |
| FCS-MTW | 0.039 | 0.053 | 0.042 | 0.031 | 0.078 | 0.063 | -0.008 | 0.078 | 0.065 | -0.081 | 0.163 | 0.129 |
| FCS-LMM | <0.001 | 0.053 | 0.044 | 0.003 | 0.079 | 0.062 | -0.001 | 0.078 | 0.065 | 0.021 | 0.152 | 0.125 |
| FCS-LMM-het | <0.001 | 0.053 | 0.042 | 0.003 | 0.079 | 0.061 | 0.004 | 0.078 | 0.066 | 0.016 | 0.151 | 0.132 |
| FCS-GLMM | <-0.001 | 0.053 | 0.045 | 0.002 | 0.079 | 0.062 | -0.002 | 0.078 | 0.068 | 0.006 | 0.152 | 0.132 |
| FCS-MLMM-LN | 0.076 | 0.052 | 0.038 | 0.059 | 0.077 | 0.056 | 0.022 | 0.078 | 0.065 | -0.052 | 0.166 | 0.137 |
| FCS-LMM-LN | 0.003 | 0.053 | 0.044 | 0.007 | 0.079 | 0.060 | 0.002 | 0.078 | 0.068 | 0.018 | 0.152 | 0.133 |
| FCS-LMM-PMM | <-0.001 | 0.053 | 0.042 | 0.004 | 0.079 | 0.061 | 0.004 | 0.078 | 0.068 | 0.019 | 0.151 | 0.132 |

| Methods | Age at wave 6 | | | Sex | | | Socio-economic position | | | Language | | |
| --- | --- | --- | --- | --- | --- | --- | --- | --- | --- | --- | --- | --- |
|  | Bias^a^ | SE^b^ | SE^c^ | Bias^a^ | SE^b^ | SE^c^ | Bias^a^ | SE^b^ | SE^c^ | Bias^a^ | SE^b^ | SE^c^ |
| Complete data | -0.005 | 0.068 | 0.068 | 0.006 | 0.028 | 0.028 | <-0.001 | 0.014 | 0.014 | -0.004 | 0.047 | 0.047 |
| Available data | <-0.001 | 0.086 | 0.088 | 0.020 | 0.036 | 0.035 | 0.022 | 0.018 | 0.018 | 0.051 | 0.063 | 0.061 |
| JM-MVN | -0.003 | 0.068 | 0.068 | 0.007 | 0.028 | 0.029 | -0.001 | 0.014 | 0.014 | -0.002 | 0.047 | 0.047 |
| JM-MLMM | -0.007 | 0.068 | 0.067 | 0.006 | 0.028 | 0.028 | -0.001 | 0.014 | 0.014 | -0.004 | 0.047 | 0.047 |
| JM-MLMM-LN | -0.003 | 0.068 | 0.067 | 0.007 | 0.028 | 0.028 | -0.001 | 0.014 | 0.014 | -0.003 | 0.047 | 0.046 |
| FCS-Standard | -0.004 | 0.068 | 0.070 | 0.006 | 0.028 | 0.027 | <-0.001 | 0.014 | 0.014 | -0.007 | 0.047 | 0.047 |
| FCS-Twofold | -0.007 | 0.068 | 0.067 | 0.007 | 0.028 | 0.029 | -0.002 | 0.014 | 0.014 | -0.004 | 0.047 | 0.047 |
| FCS-MTW | -0.005 | 0.068 | 0.068 | 0.007 | 0.028 | 0.029 | <-0.001 | 0.014 | 0.014 | -0.003 | 0.047 | 0.046 |
| FCS-LMM | -0.004 | 0.068 | 0.067 | 0.007 | 0.028 | 0.030 | -0.001 | 0.014 | 0.014 | -0.005 | 0.047 | 0.045 |
| FCS-LMM-het | -0.003 | 0.068 | 0.065 | 0.007 | 0.028 | 0.028 | <-0.001 | 0.014 | 0.014 | -0.004 | 0.047 | 0.046 |
| FCS-GLMM | -0.007 | 0.068 | 0.067 | 0.007 | 0.028 | 0.028 | -0.002 | 0.014 | 0.014 | -0.003 | 0.047 | 0.047 |
| FCS-MLMM-LN | -0.002 | 0.068 | 0.070 | 0.007 | 0.028 | 0.028 | <-0.001 | 0.014 | 0.014 | -0.004 | 0.047 | 0.047 |
| FCS-LMM-LN | -0.005 | 0.068 | 0.069 | 0.006 | 0.028 | 0.028 | <-0.001 | 0.014 | 0.014 | -0.006 | 0.047 | 0.045 |
| FCS-LMM-PMM | -0.002 | 0.068 | 0.069 | 0.006 | 0.028 | 0.029 | <-0.001 | 0.014 | 0.014 | -0.004 | 0.047 | 0.048 |

|  | Number of waves the participants lives with a single parent | | | | | | | | | | | |
| --- | --- | --- | --- | --- | --- | --- | --- | --- | --- | --- | --- | --- |
| Methods | 1 | | | 2 | | | 3-4 | | | 5 | | |
|  | Bias^a^ | SE^b^ | SE^c^ | Bias^a^ | SE^b^ | SE^c^ | Bias^a^ | SE^b^ | SE^c^ | Bias^a^ | SE^b^ | SE^c^ |
| Complete data | -0.010 | 0.057 | 0.056 | <0.001 | 0.075 | 0.078 | <-0.001 | 0.058 | 0.060 | <0.001 | 0.055 | 0.054 |
| Available data | -0.030 | 0.072 | 0.072 | -0.029 | 0.098 | 0.099 | -0.057 | 0.078 | 0.076 | -0.085 | 0.077 | 0.079 |
| JM-MVN | -0.006 | 0.061 | 0.054 | -0.027 | 0.083 | 0.070 | -0.033 | 0.062 | 0.054 | -0.004 | 0.056 | 0.055 |
| JM-MLMM | -0.005 | 0.06 | 0.054 | -0.024 | 0.083 | 0.071 | -0.023 | 0.062 | 0.056 | 0.002 | 0.056 | 0.053 |
| JM-MLMM-LN | -0.009 | 0.062 | 0.053 | 0.008 | 0.082 | 0.066 | -0.003 | 0.061 | 0.054 | 0.003 | 0.056 | 0.055 |
| FCS-Standard | 0.029 | 0.061 | 0.052 | -0.025 | 0.083 | 0.071 | -0.014 | 0.062 | 0.058 | 0.005 | 0.056 | 0.054 |
| FCS-Twofold | 0.092 | 0.06 | 0.048 | 0.095 | 0.079 | 0.066 | 0.036 | 0.061 | 0.053 | -0.061 | 0.061 | 0.054 |
| FCS-MTW | 0.092 | 0.06 | 0.048 | 0.096 | 0.080 | 0.065 | 0.035 | 0.061 | 0.056 | -0.063 | 0.061 | 0.056 |
| FCS-LMM | -0.005 | 0.06 | 0.054 | -0.019 | 0.083 | 0.072 | -0.021 | 0.062 | 0.058 | 0.002 | 0.056 | 0.053 |
| FCS-LMM-het | -0.050 | 0.061 | 0.057 | 0.026 | 0.080 | 0.068 | -0.057 | 0.063 | 0.059 | 0.012 | 0.054 | 0.054 |
| FCS-GLMM | 0.065 | 0.058 | 0.050 | 0.101 | 0.074 | 0.066 | 0.066 | 0.053 | 0.048 | -0.211 | 0.072 | 0.064 |
| FCS-MLMM-LN | 0.008 | 0.061 | 0.049 | 0.015 | 0.082 | 0.066 | 0.004 | 0.062 | 0.054 | -0.014 | 0.058 | 0.056 |
| FCS-LMM-LN | -0.016 | 0.061 | 0.050 | 0.007 | 0.081 | 0.069 | -0.007 | 0.061 | 0.056 | 0.001 | 0.056 | 0.052 |
| FCS-LMM-PMM | -0.011 | 0.061 | 0.053 | -0.009 | 0.083 | 0.069 | -0.012 | 0.061 | 0.057 | 0.007 | 0.055 | 0.054 |

^a^ Average of (estimated) bias over 1000 datasets.

^b^ Average of (estimated) model based standard errors over 1000 datasets.

^c^ Empirical standard error from 1000 estimated regression coefficients.

SEP – socio-economic status at baseline

Table S2: Average bias and standard errors in the estimated regression parameters under analysis model (2).

| Methods | BMI-z-score | | | Age | | | Sex | | | Socio-economic position | | | Language | | | Family Structure | | |
| --- | --- | --- | --- | --- | --- | --- | --- | --- | --- | --- | --- | --- | --- | --- | --- | --- | --- | --- |
|  | Bias^a^ | SE^b^ | SE^c^ | Bias^a^ | SE^b^ | SE^c^ | Bias^a^ | SE^b^ | SE^c^ | Bias^a^ | SE^b^ | SE^c^ | Bias^a^ | SE^b^ | SE^c^ | Bias^a^ | SE^b^ | SE^c^ |
| Complete data | <-0.001 | 0.006 | 0.006 | <-0.001 | 0.001 | 0.001 | <0.001 | 0.022 | 0.022 | <-0.001 | 0.011 | 0.011 | <-0.001 | 0.036 | 0.036 | <0.001 | 0.018 | 0.018 |
| Available data | -0.016 | 0.006 | 0.006 | -0.030 | 0.001 | 0.001 | 0.005 | 0.019 | 0.020 | 0.014 | 0.009 | 0.009 | 0.029 | 0.033 | 0.032 | -0.036 | 0.019 | 0.018 |
| JM-MVN | <-0.001 | 0.007 | 0.007 | <0.001 | 0.001 | 0.001 | <-0.001 | 0.021 | 0.021 | <-0.001 | 0.011 | 0.010 | <0.001 | 0.036 | 0.035 | -0.003 | 0.021 | 0.021 |
| JM-MLMM | <0.001 | 0.007 | 0.007 | <0.001 | 0.001 | 0.001 | <-0.001 | 0.021 | 0.021 | <-0.001 | 0.011 | 0.010 | 0.001 | 0.036 | 0.036 | -0.002 | 0.020 | 0.019 |
| JM-MLMM-LN | <-0.001 | 0.007 | 0.007 | <0.001 | 0.001 | 0.001 | <0.001 | 0.021 | 0.021 | <-0.001 | 0.010 | 0.011 | <0.001 | 0.036 | 0.035 | <0.001 | 0.019 | 0.020 |
| FCS-Standard | <-0.001 | 0.007 | 0.007 | <0.001 | 0.001 | 0.001 | -0.001 | 0.021 | 0.020 | <0.001 | 0.011 | 0.011 | -0.003 | 0.036 | 0.037 | <-0.001 | 0.021 | 0.022 |
| FCS-Twofold | -0.007 | 0.007 | 0.007 | <0.001 | 0.001 | 0.001 | <0.001 | 0.021 | 0.022 | -0.003 | 0.010 | 0.011 | 0.002 | 0.036 | 0.036 | -0.033 | 0.021 | 0.020 |
| FCS-MTW | -0.006 | 0.007 | 0.007 | <0.001 | 0.001 | 0.001 | <-0.001 | 0.021 | 0.020 | -0.002 | 0.011 | 0.010 | 0.001 | 0.036 | 0.036 | -0.033 | 0.021 | 0.020 |
| FCS-LMM | 0.001 | 0.007 | 0.007 | <0.001 | 0.001 | 0.001 | <-0.001 | 0.021 | 0.023 | <-0.001 | 0.011 | 0.010 | <-0.001 | 0.036 | 0.035 | -0.002 | 0.020 | 0.020 |
| FCS-LMM-het | 0.001 | 0.007 | 0.007 | <0.001 | 0.001 | 0.001 | <0.001 | 0.021 | 0.021 | -0.004 | 0.011 | 0.010 | -0.003 | 0.036 | 0.035 | -0.085 | 0.024 | 0.015 |
| FCS-GLMM | 0.002 | 0.007 | 0.007 | 0.001 | 0.001 | 0.001 | <0.001 | 0.021 | 0.021 | -0.009 | 0.011 | 0.011 | 0.003 | 0.036 | 0.035 | -0.156 | 0.021 | 0.018 |
| FCS-MLMM-LN | -0.015 | 0.008 | 0.006 | <0.001 | 0.001 | 0.001 | <0.001 | 0.021 | 0.021 | -0.002 | 0.011 | 0.010 | <-0.001 | 0.036 | 0.035 | -0.036 | 0.020 | 0.017 |
| FCS-LMM-LN | <0.001 | 0.007 | 0.007 | <-0.001 | 0.001 | 0.001 | <0.001 | 0.021 | 0.022 | <0.001 | 0.011 | 0.011 | -0.002 | 0.036 | 0.034 | -<0.001 | 0.020 | 0.020 |
| FCS-LMM-PMM | <0.001 | 0.007 | 0.007 | <0.001 | 0.001 | 0.001 | <-0.001 | 0.021 | 0.022 | -<0.001 | 0.011 | 0.010 | 0.002 | 0.036 | 0.036 | -0.021 | 0.020 | 0.019 |

^a^ Average of (estimated) bias over 1000 datasets.

^b^ Average of (estimated) model based standard error over 1000 datasets.

^c^ Empirical standard error from 1000 estimated regression coefficients.


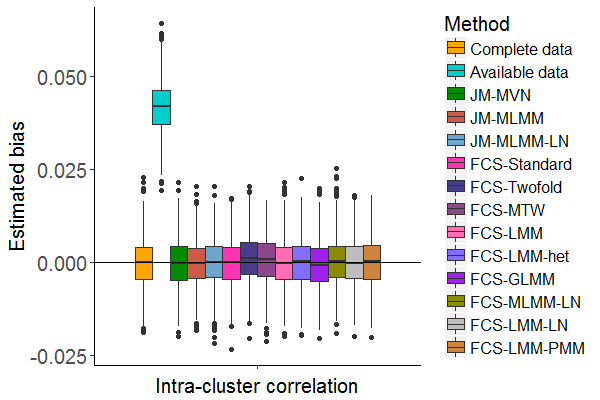


Figure S1: Distribution of estimated intra-cluster correlation coefficients for analysis model (2) across the 1000 simulated datasets.
